# Vitamin D_3_-Loaded Nanostructured Lipid Carriers as a Potential Approach for Fortifying Food Beverages; *in Vitro* and *in Vivo* Evaluation

**DOI:** 10.15171/apb.2017.008

**Published:** 2017-04-13

**Authors:** Maryam Mohammadi, Akram Pezeshki, Mehran Mesgari Abbasi, Babak Ghanbarzadeh, Hamed Hamishehkar

**Affiliations:** ^1^Biotechnology Research Center and Student Research Committee, Tabriz University of Medical Sciences, Tabriz, Iran.; ^2^Department of Food Science and Technology, Faculty of Agriculture, University of Tabriz,Tabriz, Iran.; ^3^Research Center for Pharmaceutical Nanotechnology, Tabriz University of Medical Sciences,Tabriz, Iran.; ^4^Drug Applied Research Center, Tabriz University of Medical Sciences, Tabriz, Iran.

**Keywords:** Nanostructured lipid carriers, NLC, Nanoparticle, Vitamin D3

## Abstract

***Purpose:*** Nanostructured lipid carriers (NLCs) composed of solid lipid and oil are a new generation of lipid nanoparticles which have exhibited some merits over traditional used lipid nanoparticles in fortifying food and beverages and nutraceuticals delivery systems such as liposomes and solid lipid nanoparticles.

***Methods:*** In this study, Precirol and Compritol as solid lipids, Miglyol and Octyloctanoat as liquid lipids, Tween80, Tween20 and Poloxamer407 as surfactants were used to prepare vitamin D_3_-loaded NLC dispersion using hot homogenization method. The particle size and size distribution for all formulations were evaluated by immediately after production and during a storage period of 60 days.

***Results:*** The Precirol-based NLC showed superiority over Compritol-based NLC in the point of physical stability. Results clearly suggested that an optimum concentration of 3% of Poloxamer407 or 2% of Tween20 was sufficient to cover the surface of nanoparticles effectively and prevent agglomeration during the homogenization process. Octyloctanoat was introduced for the first time as a good substituent for Miglyol in the preparation of NLC formulations. The vitamin D_3_ Intestinal absorption enhanced by the incorporating in NLCs.

***Conclusion:*** It was concluded that NLC showed a promising approach for fortifying beverages by lipophilic nutraceuticals such as vitamin D.

## Introduction

Bioactive ingredients are often enriched in functional foods to make enviable health profits. The effectiveness of these nutraceuticals, however, is diminished in some cases by their poor water solubility, instability and low bioavailability.^1^ Vitamin D is a lipophilic vitamin, acting as a seco-steroid hormone, playing a key role in the matrix of cartilage and bones. Adjusting the serum calcium and phosphorus levels within narrow limits is the main biological function of vitamin D_3_ which is important for proper bone mineralization. Furthermore, it has been indicated that vitamin D_3_ constructively engages in the functioning of the pancreas, fetal development, neural function and immunity.^2^ D_3_ is provided by the skin following sun exposure and diet in a suggested daily dose of 200-400 international units (5-10 µg) for an adult.^3^ There is a wealth of data reporting the deficiency of vitamin D in many countries around the world. Hydrophobic vitamins like vitamin D_3_cannot be dispersed in aqueous mediums (most of beverages) and are sensitive to oxidation, hence the fact that encapsulation might provide a proper way to preserve their potency and an appropriate aqueous dispersion to fortify beverages.^4,5^ Nanoparticles play an invaluable part in food industries owing to their ability to encapsulate fat-soluble nutraceuticals in order to prepare stable and transparent fortified beverages besides their enhanced oral bioavailability. Nanoparticles can generally be classified into two main groups: polymer- and lipid-based systems.^6^ The investigations on the polymer-based nanoparticles are limited by the potential toxicity of polymers,^7,8^ the limited suitable biopolymers and the obligation to use organic solvents.^9^ So as to solve these problems associated with polymeric nanoparticles, lipids have been introduced as alternative carriers, mainly of lipophilic compounds. Most nutraceuticals such as fatty acids, carotenoids, tocopherols, flavonoids, polyphenols, phytosterols and oil soluble vitamins have a lipophilic nature. Traditional lipid-based formulations include a broad range of lipid solutions, emulsions, liposomes, lipid microparticles and nanoparticles.^10^ In the early 1990s, solid lipid nanoparticles (SLNs) were introduced as an alternative drug delivery system to the existing conventional carriers, like emulsions, liposomes and polymeric nanoparticles.^11^ SLN merges the advantages of polymeric nanoparticles such as controlled drug release and drug leakage prevention which increases the delivery efficiency. Moreover, it combines the advantages of emulsion and liposome, namely high bioavailability and suitable biocompatibility, thereby decreasing the potential for acute and chronic toxicity.^12^ However, drug leakage and the inadequate loading capacity of SLN led to the development of a second, improved generation of lipid nanoparticles, namely nanostructured lipid carrier (NLC)^13^ which is a mixture of solid lipids and fluid lipids. The addition of liquid lipids causes a disruption in the ordered crystal pattern of solid lipid matrix, which, in turn enhances drug loading capacity and reduces drug leakage during storage, providing more flexibility for drug release modulation.^14^ Therefore, it seems that NLC, while not having the drawbacks of SLN, concurrently has the profits of SLN and other lipid nanocarriers. Accordingly, NLC can improve chemical stability and bioavailability of a lipophilic nutraceutical through its encapsulation and dispersion in aqueous media and the concomitant fortification of food products like beverages.^15-17^ According to our literature review, there has been no report as to the formulation of vitamin D_3_-loaded NLC until now. Moreover, in the present research, Octyloctanoat was introduced for the first time as a food grade oil phase in the development of NLC. In order to prepare a stable vitamin D_3_ loaded NLC, we extensively investigated the effect of different oils and surfactants and their mixtures on the formation and characterization of NLC. A comparative *in vivo* study was then performed on rats by orally administering vitamin D_3_ -NLC and vitamin D_3_-diluted in Miglyol oil and measuring vitamin D_3_ plasma concentration in order to evaluate the possible superiority of nanoparticulate-based formulation to conventional formulation.

## Materials and Methods

### Materials 

Precirol-ATO 5 (Glyceryldistearate) and Compritol 888 ATO (Glyceryldibehenate) were kindly donated by Gattefossè (Saint PeriestCedex, France). Miglyol-812 (caprylic/caprictriglycerides) was provided by Sasol (Witten, Germany). Tween80 and Tween20 were supplied by Merck (Hohenbrunn, Germany) and Scharlau (Sentmenat, Spain), respectively. Vitamin D_3_ standard was purchased from DSM Company (Switzerland) and Poloxamer407 and octyloctanoat was provided by Sigma Aldrich (Steinheim, Germany).

### Methods

#### Preparation of NLC

Hot homogenization technique was applied to produce NLC dispersions.^17^ Briefly, vitamin D_3_ was dissolved in liquid lipid (Miglyol) and the mixture was added to melted solid lipid (Precirol or Compritol). Next, the hot aqueous surfactant solution (with the same temperature as lipids melted mixture) was added drop-wise to the lipid phase under homogenization (Silent crusher M, Heidolph, Nuremberg, Germany) at 20000 rpm for 45 minutes. The hot o/w nanoemulsion was cooled down to room temperature resulting in lipid phase recrystallization and the ultimate formation of NLC. As listed in Table 1 we prepared various formulations with different lipids and surfactants of different concentrations, either alone or in combination with one another.

#### Characterization 

#### Scanning electron microscopy

The shape of the particles was studied via scanning electron microscope (TEscan, VEGA II XMU, Czech Republic). Prior to scanning, the samples were coated with a thin layer of gold, using a direct current sputter technique (DST1, Nanostructured coating co., Iran).

#### Particle size analysis

The size distribution of vitamin D_3_ loaded NLC was measured at 25°C with a laser light scattering technique using Particle Size Analyzer (Wing SALD 2101, Shimadzo, Japan). The intensity of the laser scattered by the samples was detected at an angle of 90°. The NLC dispersion was diluted with distilled water until suitable obscuration to prevent multiple scattering phenomena caused by inter-particle interaction. The size of each batch was measured in triplicate without the aid of pre-measurement sonication. The data related to the obtained particle size were assessed using volume distribution as diameter (D) values of D_10%_, D_50%_, D_90%_ and Span value. The diameter D_N%_ values indicate the percentage of particles possessing a diameter equal to or lower than the given value. The Span value is a helpful index to assess the particle size distribution and is calculated applying the following equation.^18^

$$Span\;=\;\frac{D_{90\%\;}-\;D_{10\%}}{D_{50\%}}$$

#### Stability study

The stability of the NLCs was investigated by measuring the particle mean diameter and span value. Three samples from each formulation were stored at ambient temperature for a period of 60 days.

#### In vivo study 

Male Wistar rats (220–260 g; Pasteur Institute of Iran, Tehran), housed in the animal facilities of

Drug Applied Research Center, Tabriz University of Medical Sciences, were kept at a controlled temperature and humidity with 12 h light: dark cycles, and had free access to water and regular rat food with the aim of specifying the oral bioavailability of the optimized formulation (F3). The animals were randomly divided into two groups (n = 6), one receiving vitamin D_3_ diluted in Miglyol oil, the other receiving vitamin D_3_ NLC (F3). The rats in both groups were given a gavage dose of 2500 IU vitamin D_3_. Blood samples (0.5 ml) of each animal were collected via the suborbital vein at 0, 0.5, 1, 2, 3, 4, 12, 24, 48, 72, 96 and 120 hours following the administration. The samples were, then, analyzed by enzyme-linked immunosorbent assays (ELISAs) for 25-Hydroxyvitamin D Total (DIAsource®, Nivelles, Belgium) using the manufacturer’s recommended protocol. The optical density was read with a microplate reader (Stat Fax-2100, Awareness TechnologyInc, Palm City, FL) at 450 and 650 nm as a reference. It should be noted that this study was conducted in accordance with the Guidelines of the Care and Use of Laboratory Animals of Tabriz University of Medical Sciences, Tabriz-Iran (National Institutes of Health Publication No. 85-23, revised 1985).

**Table 1:** Composition of Vitamin D_3_-loaded nanostructured lipid carriers

**Formulation**	**Solid Lipid (w/v)**	**Liquid Lipid (w/v)**	**Surfactant (w/v)**
F1	Precirol (4%)	Miglyol (0.4%)	Poloxamer (1%)
F2	Precirol (4%)	Miglyol (0.4%)	Poloxamer (2%)
F3	Precirol (4%)	Miglyol (0.4%)	Poloxamer (3%)
F4	Precirol (4%)	Miglyol (0.4%)	Poloxamer (4%)
F5	Precirol (4%)	Miglyol (0.4%)	Poloxamer (6%)
F6	Precirol (3.52%)	Miglyol (0.88%)	Poloxamer (3%)
F7	Precirol (2.92%)	Miglyol (1.48%)	Poloxamer (3%)
F8	Precirol (4%)	Octyl (0.4%)	Poloxamer (3%)
F9	Compritol (4%)	Miglyol (0.4%)	Poloxamer (1%)
F10	Compritol (4%)	Miglyol (0.4%)	Poloxamer (2%)
F11	Compritol (4%)	Miglyol (0.4%)	Poloxamer (3%)
F12	Compritol (4%)	Miglyol (0.4%)	Poloxamer (4%)
F13	Compritol (4%)	Miglyol (0.4%)	Poloxamer (6%)
F14	Compritol (3.52%)	Miglyol (0.88%)	Poloxamer (3%)
F15	Compritol (2.92%)	Miglyol (1.48%)	Poloxamer (3%)
F16	Compritol (4%)	Octyl (0.4%)	Poloxamer (3%)
F17	Precirol (4%)	Miglyol (0.4%)	Tween 20 (1%)
F18	Precirol (4%)	Miglyol (0.4%)	Tween 20 (2%)
F19	Precirol (4%)	Miglyol (0.4%)	Tween 20 (3%)
F20	Precirol (4%)	Miglyol (0.4%)	Tween 20 (4%)
F21	Precirol (4%)	Miglyol (0.4%)	Tween 20 (6%)
F22	Precirol (3.52%)	Miglyol (0.88%)	Tween 20 (2%)
F23	Precirol (2.92%)	Miglyol (1.48%)	Tween 20 (2%)
F24	Precirol (4%)	Miglyol (0.4%)	Tween 20 (1%) + Poloxamer (1%)
F25	Precirol (4%)	Octyl (0.4%)	Tween 20 (2%)
F26	Precirol (4%)	Miglyol (0.4%)	Tween 80 (1%)
F27	Precirol (4%)	Miglyol (0.4%)	Tween 80 (2%)
F28	Precirol (4%)	Miglyol (0.4%)	Tween 80 (3%)
F29	Precirol (4%)	Miglyol (0.4%)	Tween 80 (4%)
F30	Precirol (4%)	Miglyol (0.4%)	Tween 80 (6%)
F31	Precirol (3.52%)	Miglyol (0.88%)	Tween 80 (2%)
F32	Precirol (2.92%)	Miglyol (1.48%)	Tween 80 (2%)
F33	Precirol (4%)	Miglyol (0.4%)	Tween 80 (2%) + Poloxamer (1%)
F34	Precirol (4%)	Miglyol (0.4%)	Tween 80 (2%) + Poloxamer (2%)
F35	Precirol (4%)	Octyl (0.4%)	Tween 80 (2%)

#### Statistical analysis

Each value was expressed as the mean ± standard deviation. Statistical analysis was performed using a one-way analysis of variance (one-way ANOVA) with multiple comparisons between deposition data using a Tukey honest significant difference test (SPSS, version 13.0, Chicago, IL, USA). A P_value_ of <0.05 was considered significant.

## Results and Discussion

### Preparation of vitamin D_3_loaded NLC

**Figure 1:**
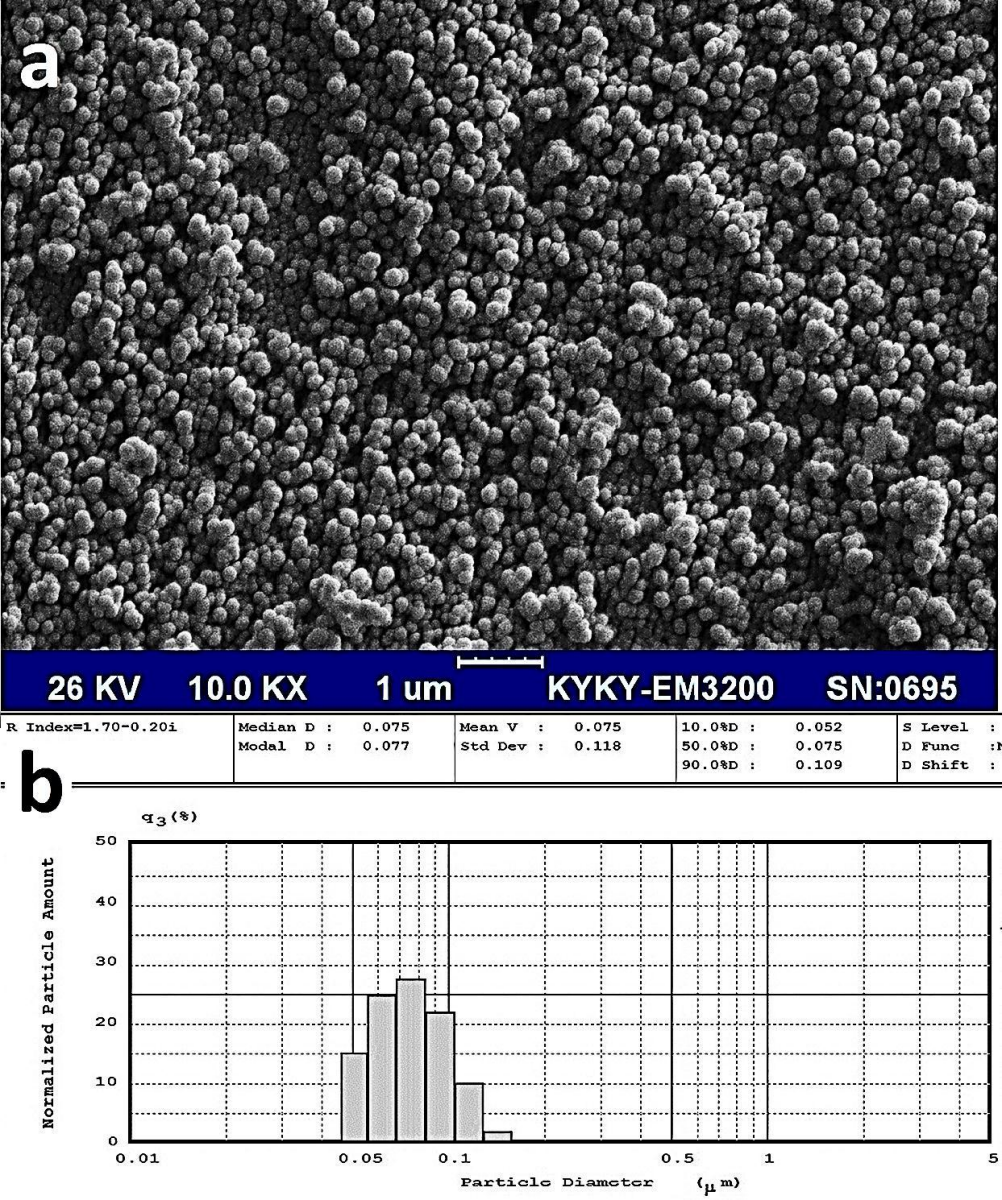
Scanning electron micrograph (a) and size pattern (b) of formulation F3 (optimized formulation).

Scanning electron micrographs (SEM) of formulation F3 (optimized formulation) indicated that Vit D_3_-loaded NLC were successfully prepared (Figure 1a) with a relatively uniform size distribution (Figure 1b).

### Particle size measurement

Size characteristics of samples are tabulated in Table 2. Particle size results indicate that the size and size distribution of the formulations were greatly dependent on the formulation compositions (type and amount of lipids and surfactants) and varied between 77 nm (F3) and 2504nm (F30).

A wide range of solid lipids and liquid lipids including natural, semisynthetic and synthetic lipids with various structures like that of triglycerides, partial glycerides, fatty acids, waxes and steroids can be used as matrix lipids for NLC preparation.^19^ Toxicity and biocompatibility seem to be the two most important issues to be considered in lipid selection for NLC fabrication for use in food industries.^17,20^ The administered lipids in this study, namely glyceryl dibehenate (Compritol), glyceryl distearate (Precirol), caprylic/capric triglycerides (Miglyol) and Octyloctanoat have been approved as generally recognized as safe (GRAS) for direct addition to food.^11^

### The effect of surfactant types and concentrations on the particle size

**Figure 2:**
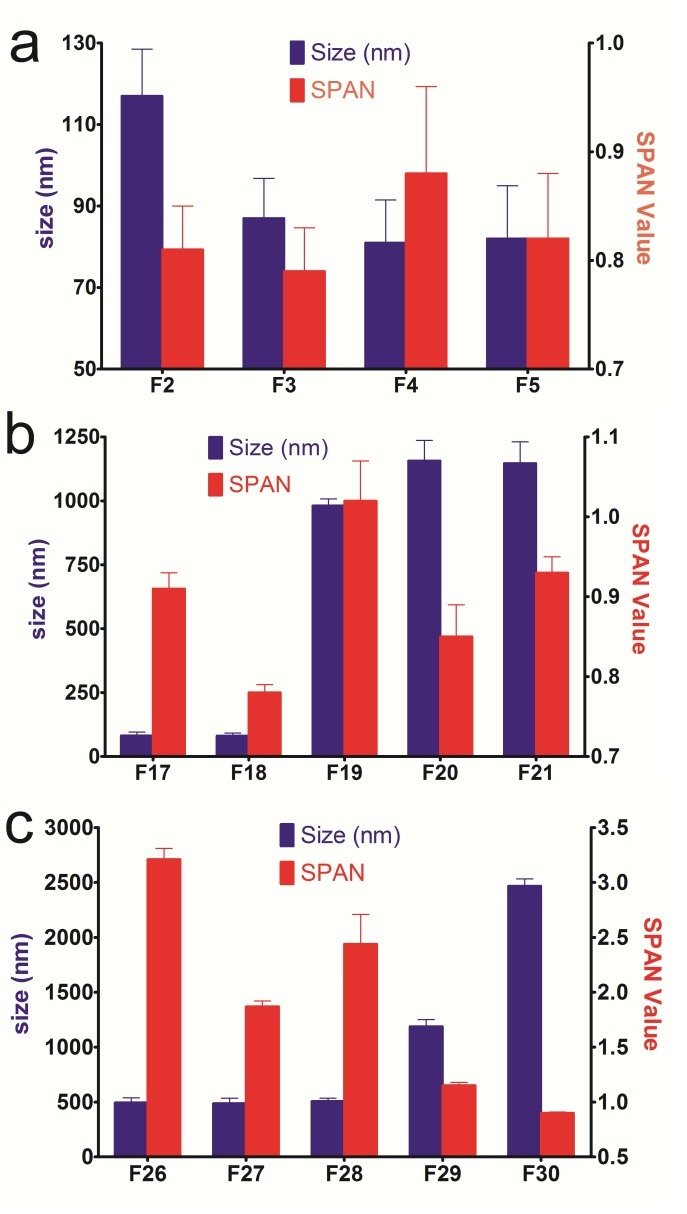
The impact of increasing concentrations of Poloxamer (a), Tween20 (b) and Tween80 (c) on the particle size and size distribution of NLC formulations.

The primary role of a surfactant is to stabilize nanoparticles in the colloidal state and prevent particle aggregation during storage. The choice of stabilizers is the key to preparing any nanoparticle formulation as they control the particle size and the stabilization of the dispersions.^21^Poloxamer is a hydrophilic non-ionic surfactant, blocking the copolymer of polyethylene oxide (PEO) and polypropylene oxide (PPO). The hydrophobic PPO chains are adsorb on the particle surfaces as the “anchor chain”, while the hydrophilic PEO chains are pulled out from the surface to the aqueous medium, thereby creating a stabilizer layer.^22^ The impact of a Poloxamer concentration increasing from 1% (w/w) to 6% (w/w) was also studied on the particle size and physical stability (Figure 2a).

**Table 2:** Size characteristics of VitD_3_-loaded nanostructured lipid carriers (mean ± SD, n= 3)

**Formulation**	**Cumulative percent (undersize)** ^a^			**VMD** ^b^ **(nm)**	**Span Value**
	**D10% (nm)**	**D50% (nm)**	**D90% (nm)**		
F1	ND^c^	ND	ND	ND	ND
F2	59 ± 5.9	85 ± 10.4	126 ± 12.2	117± 11.5	0.81 ± 0.04
F3	64 ± 8.7	88 ± 13.6	133 ± 11.3	87 ± 9.8	0.79 ± 0.04
F4	59 ± 8.5	83 ± 11.8	119 ± 9.7	81 ± 10.5	0.88 ± 0.08
F5	63 ± 9.1	81 ± 10.4	124 ± 14.4	82 ± 13	0.82 ± 0.06
F6	61 ± 17.5	87 ± 14.2	151 ± 14	90 ± 15.9	0.93 ± 0.15
F7	106 ± 12.1	357 ± 35.2	2309 ± 67.4	475 ± 18	3.65 ± 0.19
F8	60 ± 7.5	83 ± 12.2	123 ± 15.3	88 ± 13.9	0.77 ± 0.01
F9	540 ± 30	1112 ± 83.1	2363 ± 71	1010 ± 36.1	1.78 ± 0.07
F10	280 ± 26.5	492 ± 36.9	1017 ± 35.1	503 ± 25.2	1.39 ± 0.03
F11	64 ± 9.3	86 ± 11.5	125 ± 14.5	82 ± 11.7	0.89 ± 0.04
F12	62 ± 8.7	90 ± 16.6	125 ± 25.2	86 ± 17.2	0.94 ± 0.07
F13	77 ± 7.1	106 ± 14	147 ± 13.9	219 ± 30.2	1.23 ± 0.07
F14	61 ± 5.9	80 ± 10.6	150 ± 13.2	86 ± 9.5	0.90 ± 0.02
F15	272 ± 20.2	446 ± 31.9	125 ± 30.1	461 ± 36.4	1.17 ± 0.01
F16	58 ± 8.3	86 ± 9.6	818 ± 15	86 ± 18.3	0.9 ± 0.09
F17	61 ± 7.6	87 ± 13.3	125 ± 18	82 ± 13.6	0.91 ± 0.02
F18	58 ± 9	82 ± 14.5	115 ± 8.7	81 ± 11	0.78 ± 0.01
F19	596 ± 41.5	948 ± 53.5	1623 ± 50.3	981 ± 27	1.02 ± 0.05
F20	766 ± 4.7	1190 ± 98.5	1767 ± 50	1157 ± 79.8	0.85 ± 0.04
F21	731 ± 46.5	1217 ± 45.1	1815 ± 49.3	1147 ± 84.1	0.93 ± 0.02
F22	67 ± 8.1	101 ± 12.1	153 ± 12.5	85 ± 10.1	0.83 ± 0.02
F23	62 ± 9.7	86 ± 11.3	137 ± 25.2	85 ± 17	0.78 ± 0.03
F24	59 ± 7.8	84 ± 13	125 ± 15	84 ± 12.6	0.90 ± 0.01
F25	59 ± 7.8	83 ± 12.9	130 ± 15	82 ± 13.1	0.80 ± 0.01
F26	226 ± 31.7	510 ± 30	1303 ± 41.6	495 ± 47.6	3.21 ± 0.01
F27	228 ± 36.6	445 ± 45	1081 ± 70	488 ± 43	1.87 ± 0.05
F28	243 ± 40.4	443 ± 43.5	1022 ± 35.4	508 ± 27.1	2.44 ± 0.57
F29	1026 ± 48.3	1533 ± 54	2337 ± 51.4	1188 ± 62.9	1.15 ± 0.27
F30	1555 ± 42.7	2473 ± 66.6	3822 ± 85.7	2470 ± 62	0.90 ± 0.01
F31	320 ± 30	443 ± 48.6	849 ± 44	480 ± 26.8	1.13 ± 0.15
F32	275 ± 31.2	419 ± 35.6	638 ± 40.4	459 ± 27.2	0.86 ± 0.04
F33	59 ± 8.1	80 ± 9.2	119 ± 10.3	81 ± 10.1	0.74 ± 0.03
F34	63 ± 8.7	88 ± 8.7	126 ± 15.2	92 ± 17.8	0.66 ± 0.11
F35	145 ± 23.8	342 ± 35.2	661± 53	350 ± 43.5	1.51 ± 0.7

^a^ Equivalent volume diameters at 10 (D_10%_), 50 (D_50%_) and 90% (D_90%_) cumulative volume. ^b^ Volume median diameter. ^c^ Not determined.

Unstable and spontaneous gel formation as well as particle growth and agglomeration were observed for F1 within one hour of storage at 25°C. Gel formation takes place by the NLC particles building up a network and bridging among lipid particles due to inefficient surfactant performance. Like coalescence, gel formation is an irreversible progression leading to the loss of the colloidal system.^9^The particles prepared with 2% Poloxamer (F2) had a small particle size (117 nm) with suitable size distribution (span value 0.78) indicating the homogenous nature of the dispersion. The formulation grew in size from 117 nm to 373 nm after a month and turned into gel following two months of storage. This may be attributed to the formation of new hydrophobic surfaces upon crystallization due to the change in the shape of the particles, leading to aggregation through non-polar patches on different particles, which requires that additional surfactants be covered.^23^ Unlike Poloxamer, other surfactants formed stable NLC dispersions in 2% of the concentration (w/v) after two months of storage. This might be related to its large structure (with a molecular weight of around 10000-14000 g/mol compared to Tween 80 and 20 which are 1310 and 1225, respectively). If, in covering the exposed hydrophobic patches, particle–particle collisions occur faster than surfactant molecule absorption, extensive particle aggregation will occur due to hydrophobic attraction between the particles.^24^ Due to its huge structure, Poloxamer was probably not able to promptly cover the newly formed lipid particles and be absorbed to the surface of lipidic particles as rapidly as the rate of particle–particle collisions. The particle sizes of vitamin D_3_-loaded NLC stabilized with 3%, 4% and 6% Poloxamer were almost the same (no significant difference; P˂0.05), having a mean diameter of 78-95 nm (small size) and a span value of 0.73-0.79 (narrow size distribution). Furthermore, all formulations were physically stable over the two-month studied period (Table 3). The results suggested that an optimum Poloxamer concentration (i.e.3%) was sufficient to effectively cover the surface of nanoparticles and prevent agglomeration during the homogenization process although there was no statistical difference among the results of 3, 4 and 6 % Poloxamer. The influence of the Poloxamer on particle size and span value of NLC formulation composed of a different solid lipid (Compritol) was also examined (F9 –F13). Although Poloxamer with the concentration of 1% was able to prepare an emulsion (with a large size of around 1 µm), it turned to gel after two months, an instability which was also the case with F10. Through Compritol, the remaining formulations (F11-F16) were compared in size with their identical formulation compositions prepared with Precirol (F3-F8); no statistical difference was observed,, indicating that the type of solid lipid (Compritol and Precirol) did not affect the size of Vitamin D_3_-loaded NLC. However, after two months of storage, Precirol-made NLCs had a better size stability than NLCs composed of Compritol (Table 3).

Accordingly, Precirol was used to fabricate the rest of the formulations. In order to select a suitable lipid for the preparation of drug-loaded SLN, Liu et al., 2007, studied the suitability of different lipids, namely Compritol, Precirol and glycerin monostearate. The Precirol-based SLN showed the lowest size, size distribution and physical stability compared to other lipids. Tween20 is a non-ionic surfactant with a relatively low critical micelle concentration (0.06 mM), hence rendering the surfactant monomer concentration in the continuous phase relatively low.^11^ In this study, varying amounts of Tween20 (1-6 w/v%) were used to prepare vitamin D_3-_loaded NLC composed of Precirol (as solid lipid) and Miglyol (as oil)The effect of these concentrations were then investigated on the particle size, size distribution and physical stability (Formulations F17-F25; Table 1). The formulation prepared with 1% Tween20 (F17) exhibited a small particle size (86 nm) with a span value of 0.91, indicating a narrow size distribution and homogenous dispersion. This formulation, however, showed an extensive particle enlargement and aggregation after one month of storage and tended to form a gel following two months. Insufficient surfactant concentrations during the crystallization process may lead to particle aggregation and destabilization.F24 was prepared with the same composition of F17, except this time a secondary stabilizer Poloxamer was added. The size and size distribution results were satisfying (Table 2). The stability data was desirable until after two months of the storage (Table 3). Poloxamer has a large molecular weight and is a non-ionic block copolymer of polyoxyethylene oxide (hydrophilic section) and polyoxypropylene oxide (hydrophobic section). The hydrophobic segment accommodates at interface and a dense polymeric network of hydrophilic segment orients in the external phase.^22^ This mutual hydrophilic/hydrophobic interaction with NLC provides adequate steric hindrance to the particle growth. The comparison of F24 with F17 and F1 indicated that both surfactants help each other in producing and stabilizing NLC at the mentioned low concentrations. Previous studies have reported that combination of Poloxamer 188 (0.25%, w/v) and Tween 20 (2.5%, w/v) is optimal to prevent particle growth and stabilize the system for more than 24 hours.^25^ Polysorbate (Tween 80), often used in foods, is a nonionic surfactant and emulsifier derived from polyethoxylatedsorbitan and oleic acid.^11^ In this study, Tween80 was investigated at various concentrations (1-6% w/v) all of which showed high particle sizes ranging from around 400 nm to 2500 nm and span values ranging from 1.86 to 3.21, hence the broad and heterogeneous particle size distribution. In the presence of 2% Tween 20 (F18 and F27) smaller particle sizes and narrow size distributions were observed compared to formulations prepared with other concentrations of Tween20 (F17, F19 and F20), as was the case with Tween80. With higher concentrations of both Tweens, an increase was observed in the mean particle size and size distribution of the NLC formulations. Probably at high surfactant concentrations, the surfactant tail groups are bound to be tightly packed together at the oil–water interface resulting in more solid-like characteristics than loosely packed surfactant tails which consequently thwart the molecular motion of the triacylglycerol molecules within the droplet interior. It has already been reported that with the increase in concentration, the rigidity of Tween20 interfaces augment.^24^The rigid surfactant shell may work as a physical constraint that decelerates molecular movement within the particles, causing particle aggregation and increasing the mean particle size. Our data corroborates the administration of low concentrations of Tweens (Figure 2b,c). The observed size instability of formulation F17 (containing 1% Tween20) suggests that a minimum of 2% Tween20 is needed to saturate the emulsion droplet surfaces prior to lipid crystallization. Although at 1% concentration, Tween80 produced a stable NLC, the size was larger than the formulation composed of the same concentrations of Tween20. The larger NLC formulations produced by Tween80 compared to those fabricated by Tween20 (F17-F21) might be attributed to the production temperature (85°C), which is higher than the cloud point of the Tween80 (72.6°C) and lower than that of Tween20 (95°C). Although high temperature facilitates the break-up of droplets by lowering the viscosity and interfacial tension, it may affect the nature of the emulsifier. Above the cloud point, the hydrophilic group of the emulsifier becomes dehydrated, reducing the hydration repulsion between them, at which point, the emulsifier cannot prevent the aggregation of the emulsion droplets, leading to the formation of larger emulsion droplets.^1^ This finding is in good agreement with the reported results of NLC containing heat-sensitive bioactives (beta carotene).^26^ The size of all formulations prepared with Tween80 (F26-F30) and most formulations prepared with Tween20 (F18-F21) was stable during the storage time.. The results observed in F24 (combination of Tween20 and Poloxamer) encouraged us to prepare an identical formulation with Tween80 (F33) and a formulation with higher concentrations of Poloxamer (F34). The results indicated that all formulations had the same size. The size distribution (span value), however, was smaller for F33 and even much smaller for F34. It can be concluded that Tween80 entails a narrower size distribution than Tween20. And the increase in Poloxamer concentration, while maintaining the stability, results in a much narrower distribution. Poloxamer and other emulsifiers with large molecular weights, offer additional steric stabilization effect, preventing the aggregation of fine particles in the colloidal system. Combining two or more emulsifiers seem to produce mixed surfactant films at the interface, generating high surfactant coverage as well as adequate viscosity to improve the stability and synergism in the particle size reduction. It was also shown that the combination of Tween80 and Egg phosphatidylcholine reduced the particle size and polydispersity index of all-trans-retinoic acid-loaded SLNs.^27^

### The effect of oil types and concentrations on the particle size

**Figure 3:**
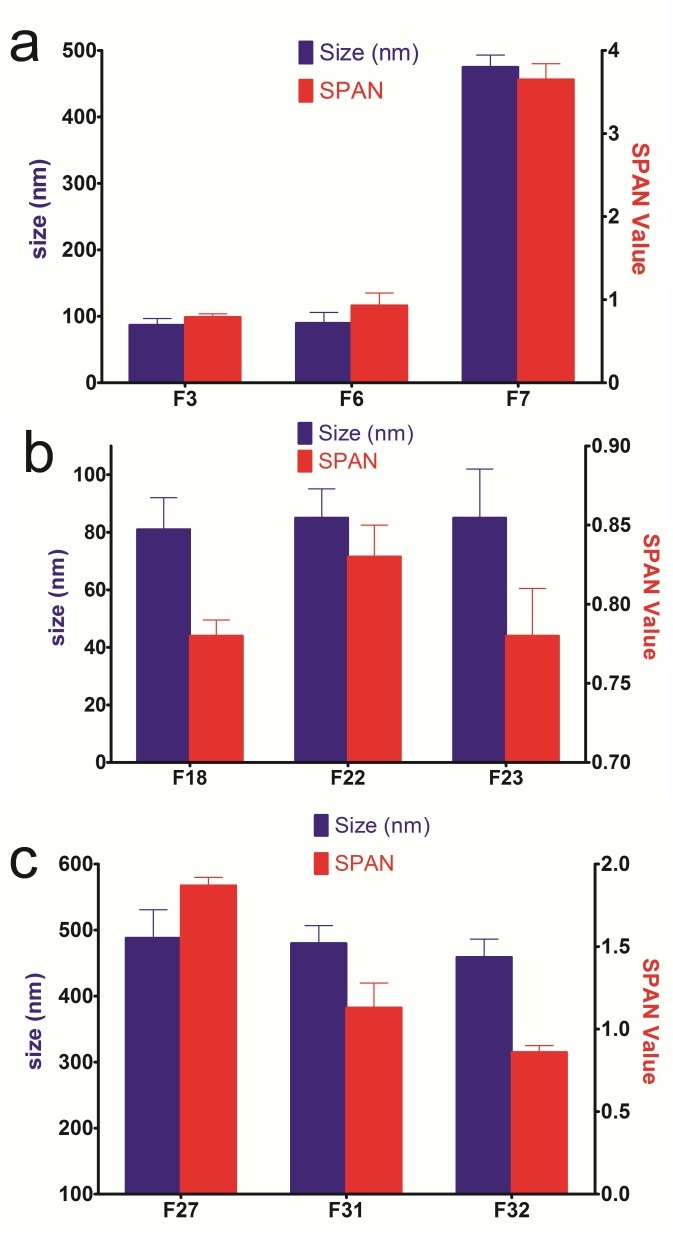
The impact of increasing concentrations of Oil to solid lipid in the presence of different surfactants; Poloxamer (a), Tween20 (b) and Tween80 (c) on the particle size and size distribution of NLC formulations.

Miglyol is a type of oil routinely used in the preparation of NLC.^11,28^ Liquid oils have been reported to play important roles in the characteristics of NLCs. In the present research, therefore, Miglyol 812 and Octyloctanoate were considered as suitable liquid oils for vitamin D_3_-loaded NLC. Miglyol, a medium chain triglyceride (MCT), is a unique class of saturated lipids composed mainly of caprylic (C8:0; 50–80%) and capric (C10:0; 20–50%) fatty acids with a minor level of caproic (C6:0; ≤ 2%), lauric (C12:0; ≤ 3%) and myristic (C14:0; ≤ 1%) fatty acids. MCT is a food-grade and odorless produced by the modification (hydrolysis/esterification) and fractionation of natural oils, such as coconut or palm kernel oils. It is known to be digested more rapidly than such long chain triglycerides as corn oil and shows high stability against oxidation. This class of lipids has been approved as generally recognized as safe (GRAS) by the US Food and Drug Administration (FDA) and permitted to be added directly to many food types (including beverages) for specific functional uses (e.g. as vehicle, solvent, release agent or emulsifier).^11,29^ According to Figure 3, the increase in the Miglyol content from 10% to 20% did not influence the mean particle size and particle size distribution of the formulations with the same composition (F3 and F6, F11 and F14, F18 and 22 and F27 and F31).

**Table 3:** Size stability evaluation of Vitamin D_3_-loaded nanostructured lipid carriers (mean ± SD, n= 3).

**Formulation**	**Month 1**		**Month 2**	
	**VMD** ^a^ ** (nm)**	**Span Value**	**VMD** ^a^ ** (nm)**	**Span Value**
F1	ND^b^	ND	ND	ND
F2	334 ± 48.4	1.75 ± 0.06	ND	ND
F3	85 ± 11.2	0.8 ± 0.05	86 ± 11.1	0.76 ± 0.05
F4	85 ± 10.5	0.88 ± 0.11	86 ± 11.5	0.81 ± 0.1
F5	82 ± 8.5	0.82 ± 0.15	87 ± 11	0.76 ± 0.06
F6	89 ± 9.5	0.78 ± 0.04	88 ± 10.5	0.85 ± 0.05
F7	708 ± 43.1	3.64 ± 0.11	743 ± 81.4	4.02 ± 0.25
F8	78 ± 10.1	0.75 ± 0.05	79 ± 9	0.72 ± 0.06
F9	1126 ± 87.9	1.87 ± 0.06	ND	ND
F10	551 ± 56.1	1.44 ± 0.02	ND	ND
F11	84 ± 8.5	0.84 ± 0.06	86 ± 9.2	0.92 ± 0.08
F12	240 ± 27.8	1.06 ± 0.14	253 ± 61.1	1.12 ± 0.1
F13	422 ± 30.3	1.4 ± 0.06	552 ± 63.5	1.45 ± 0.05
F14	348 ± 39.6	0.96 ± 0.12	360 ± 55.7	0.87 ± 0.05
F15	503 ± 74.5	0.87 ± 0.05	523 ± 58.6	1.05 ± 0.13
F16	82 ± 12.2	0.78 ± 0.02	85 ± 9.3	0.75 ± 0.05
F17	3016 ± 104	6.14 ± 0.05	ND	ND
F18	85 ± 10.3	0.77 ± 0.02	85 ± 7	0.83 ± 0.06
F19	945 ± 68	1.31 ± 0.04	976 ± 86.2	1.32 ± 0.03
F20	1174 ± 75	1.27 ± 0.07	1233 ± 76.4	1.28 ± 0.03
F21	1158 ± 87.8	1.37 ± 0.07	1257 ± 60.3	1.57 ± 0.04
F22	86 ± 11	0.83 ± 0.05	86 ± 9.3	0.82 ± 0.06
F23	86 ± 7.8	0.78 ± 0.03	88 ± 11	0.84 ± 0.1
F24	85 ± 11.6	0.86 ± 0.11	98 ± 12	1 ± 0.04
F25	78 ± 7	0.8 ± 0.02	86 ± 11.9	0.85 ± 0.09
F26	488 ± 43	1.83 ± 0.06	515 ± 35	1.83 ± 0.09
F27	486 ± 44.7	3.16 ± 0.06	510 ± 40.1	3.25 ± 0.06
F28	533 ± 61.3	2.89 ± 0.09	543 ± 60.3	2.86 ± 0.08
F29	1279 ± 77.1	1.56 ± 0.05	1326 ± 64.3	1.65 ± 0.05
F30	2504 ± 127	1.55 ± 0.13	2577 ± 75.1	1.67 ± 0.15
F31	433 ± 36.5	1.06 ± 0.05	507 ± 40.3	1.11 ± 0.09
F32	437 ± 40.2	0.92 ± 0.03	468 ± 48.1	1.05 ± 0.06
F33	86 ± 9.2	0.78 ± 0.03	88 ± 10.1	0.78 ± 0.03
F34	86 ± 9.5	0.75 ± 0.05	90 ± 8.1	0.77 ± 0.04
F35	380 ± 40	1.36 ± 0.06	410 ± 40	1.43 ± 0.06

^a^ Volume median diameter. ^c^ Not determined.

The increase up to 36% of the Miglyol to solid lipid ratio did not change the size of the NLC formulations prepared with Tween20 and Tween80, whereas the size distribution improved and grew narrower (P<0.05). Nonetheless, the size of the formulations prepared with Poloxamer (F7 and F15) increased dramatically once the oil content reached 36%. At low liquid lipid concentrations, the oil molecules are dispersed within the solid lipid matrix and an imperfect type of NLC is obtained. In this case, the presence of liquid lipid does not change the size of NLC. Muller and co-workers reported that during the cooling down process, the miscibility of the oil in the solid lipid decreased in higher oil percentages, causing phase separation.^17^ We found that Tween80 and Tween20 could dissolve in the mixture of lipids but Poloxamer precipitated (data are not shown). When, during the cooling process of NLC preparation, the oil precipitates in the form of fine droplets in the solid lipid matrix, they might be stabilized by dissolved surfactants and disperse in the form of tiny droplets without a recognizable change in the size. On the other hand, due to the insolubility of Poloxamer in the lipid phase, it is not able to stabilize the formed oil droplets in solid lipid matrix and owing to their aggregation in forming larger droplets the particle size increases. Recently, it was reported that increasing the oil content from 10 to 20% did not significantly increase the particle size (110 nm to 120 nm). Nevertheless, the particle size was considerably reduced once the oil content hit 30%, a strange observation that was not clearly explained.^27^ Although Agrawa et al., through an experimental design study, indicated that the particle size of the Acitretin-loaded NLCs (prepared with Tween80) was affected by the oleic acid content , the presented surface and counter plots did not ensue a significant change for the readers.^30^ The particle size enlargement of clotrimazole-loaded NLCs by the increase in the percentage of liquid lipid does not seem to be statistically significant either given the presented Figure.^31^ Kovasevic et al. reported results consistent with ours: The liquid oil (Miglyol) in the range of 20-60% did not influence the size of NLC.^28^

Octyloctanoate is a colorless liquid with a faint, green tea odor that has been approved as GRAS by the FDA.^11^ Due to its safety and biocompatibility, it was applied, for the first time, as an oil phase in the preparation of optimized NLC formulations (F8, F16, F25 and F35) and compared with Miglyol. The comparison of identical formulations (F3 and F8, F11 and F16, F18 and F25) indicated that the size did not change significantly when Octyloctanoate was used instead of Miglyol). The particle size distributions of the formulations prepared by Miglyol and Octyloctanoate were the same, yet different from the formulations prepared with Tween80. Moreover, formulations prepared with Tween80 had higher sizes and SPAN values than those prepared with Tween20 and Poloxamer. Octyloctanoate reduced the size and SPAN value of F27 when used, under identical conditions, instead of Miglyol. Furthermore, these formulations were stable over the two months of storage. According to the obtained results, Octyloctanoat has a potential to be introduced as a good substituent for Miglyol in the preparation of NLC formulations.

### Intestinal absorption

**Figure 4:**
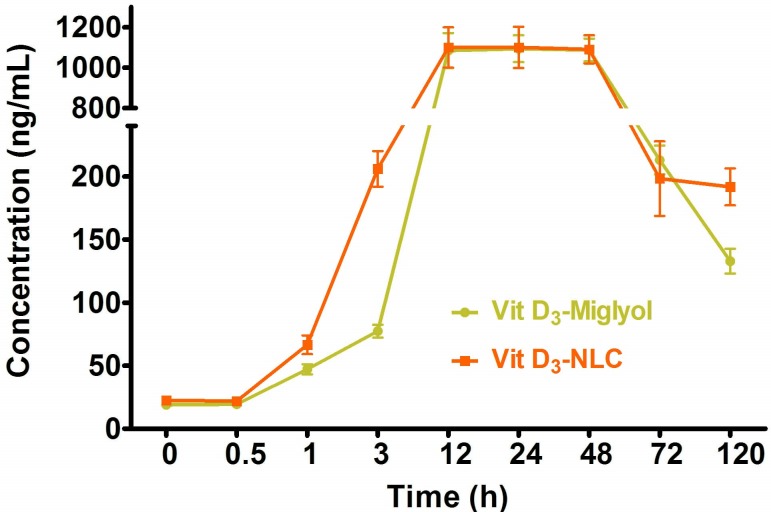
The plasma concentration–time curve of vitamin D_3_- NLCs and vitamin D_3_-diluted in Miglyol oil (data presented as mean ± standard deviation, n=6)

There are concerns as to the bioavailability, bioequivalence and exchangeability of the different formulations used in nutraceutical compounds, for which there are many factors that can change the release, dissolution and absorption of the bioactives in the body. There exist a myriad of ways to evaluate the bioavailability of bioactives, but the recommended way is to determine the bioactive quantity in body fluids (blood, plasma) as a function of time, because of its superior precision and accuracy.^32^Vitamin D_3_ NLCs and vitamin D_3_ solutions were orally administrated to male Wistar rats. The plasma concentration–time curve in Figure 4 shows that the NLC formulation presented the faster appearance of vitamin D_3_ in the plasma than the oily solution formulation. Furthermore, the NLC formulation resulted in more prolonged vitamin D_3_ plasma level.Vitamin D is a liposoluble vitamin, and its relative bioavailability could bring about unfavorable conditions.^33^

The most popular method to improve the oral bioavailability of lipophilic bioactives is to utilize ipid-based formulations. The mechanisms behind this approach include elongating GIT residence time, triggering lymphatic transport, increasing the permeability of the intestinal wall and reducing metabolism and efflux activity.^34^The improved drug bioavailability of nanoparticles is attributed to the fact that particles in nano-size ranges are efﬁcient in crossing the permeability barriers.^35^ It has been indicated that nano-particle transposition is mediated by Peyer’s patches (M cells) in the small intestine. Once these particles enter the systemic circulation through lymphatic transport, the oral drug absorption is improved. Moreover, by passing the liver through lymphatic transport ﬁrst-pass metabolism is reduced and consequently oral drug bioavailability is enhanced.^36^

## Conclusion

Selecting a proper formulation composition and amount is the key to a successful production of NLC with appropriate physical and chemical specifications. The size, size distribution and physical stability of our prepared vitamin D_3_-loaded NLCs were greatly dependent on selecting suitable types and amounts of solid lipid, oil, and surfactant, during preparation and storage. The results indicated that for a stable NLC, an optimized surfactant concentration is required. Among the three assessed non-ionic surfactants (Poloxamer407, Tween80, Tween20), Poloxamer407 entailed a stable formulation with the lowest size and particle size distribution at almost all concentrations. It was also concluded that Tween80, alone, may not be propitious as a surfactant to stabilize nanoparticles composed of vitamin D_3_. Octyloctanoat was introduced as a good substituent for Miglyol in the preparation of NLC formulations. Ultimately, the results illustrated that the incorporation of vitamin D_3_ into NLCs increased the absorption of vitamin D_3_ by oral administration.

## Acknowledgments

The authors would like to thank Drug Applied Research Center, Tabriz University of Medical Sciences for financial support. This paper was financially supported by grant no. 91/96 from the Drug Applied Research Center of Tabriz Medical Sciences University.

## Ethical Issues

Not applicable.

## Conflict of Interest

The authors have no conflicts of interest to declare.
